# Identification of hexose kinase genes in *Kluyveromyces marxianus* and thermo-tolerant one step producing glucose-free fructose strain construction

**DOI:** 10.1038/srep45104

**Published:** 2017-03-24

**Authors:** Guorong Zhang, Min Lu, Jichao Wang, Dongmei Wang, Xiaolian Gao, Jiong Hong

**Affiliations:** 1 School of Life Sciences, University of Science and Technology of China, Hefei, Anhui, 230027, P. R. China

## Abstract

In yeast, the hexose assimilation is started at hexose phosphorylation. However, in *Kluyveromyces marxianus*, the hexokinase (*HXK*) and glucokinase (*GLK*) genes were not identified by experiments. Meanwhile, the glucose-free fructose product requires more cost-efficient method. In this study, the *KmHXK1* and *KmGLK1* genes were functionally identified through gene disruption, over-expression and recombinant enzymes characterization. Both glucose and fructose assimilation ability decreased significantly in *KmHXK1* disrupted strain YLM001, however, this ability was not changed obviously in *KmGLK1* disrupted strain YLM002. When over-expressing *KmGLK1* in YLM001, only the glucose assimilation ability was recovered in obtained strain (YLM005). The kinetic constant analysis of recombinant enzymes also proved that KmHXK1 could phosphorylate glucose (*V*max 553.01 U/mg, *K*m 0.83 mM) and fructose (*V*max 609.82 U/mg, *K*m 0.52 mM), and KmGLK1 only phosphorylate glucose with a *V*max of 0.73 U/mg and a *K*m 4.09 mM. A thermo-tolerant strain YGR003 which produced glucose-free fructose from Jerusalem artichoke tuber in one step was constructed based on the obtained information. The highest production and fastest productivity were 234.44 g/L and 10.26 g/L/h, respectively, which were several folds of the results in previous reports.

In yeast the hexoses including glucose and fructose are phosphorylated by hexose-glucose kinase after uptake, then enter the glycolytic pathway. In *Saccharomyces cerevisiae*, three enzymes (encoded by *HXK1, HXK2*, and *GLK1*) can catalyze this reaction^1^. However, only one hexokinase (*KmHXK1*) and one glucokinase (*KmGLK1*) gene were found in the genome of *Kluyveromyces marxianus* based on theoretical translation and homology analysis^2^,^3^, and the functions of the two enzymes were not experimentally confirmed.

With the growing demand for natural, healthy and low-calorie food, a large number of alternative sugars has emerged since the early 1980 s^4^. Nutritive sweeteners (e.g., sucrose, fructose) are generally recognized as safe (GRAS) by the Food and Drug Administration (FDA)^5^. Other than as the highest sugar sweetener, fructose is also used as an excipient in pharmaceutical tablets, syrups and solutions. It makes medicines more palatable for the high sweetness and safety^6^,^7^. The ability to inhibit water crystallisation allows it to serve as a cryoprotectant^8^, The high solubility of fructose enables it to aid the solubility of therapeutic agents and adjusting osmolarity of solutions to make them compatible with parenteral administration^7^. Fructose is also used as lyoprotectants for its ability of protecting against the fundamentally different stresses of freezing and subsequent dehydration^9^. Also, fructose is metabolic in liver and does not require insulin, therefore it is beneficial to diabetics. Some studies showed that the consumption of high-fructose corn syrup (HFCS) or fructose may raise the risk of obesity and diabetes. However, most of them have a premise of excess consumption. Whether the fructose is the unique reason is still under contest, and more possibly, the overtake of calories overall led these epidemics^10^,^11^,^12^,^13^.

However, producing glucose-free fructose is still a costly and multistep procedure. Fructose is usually available in blends with other sugars, named as high-fructose corn syrup (HFCS)^4^ and is produced with glucose isomerase from corn starch hydrolysate. The demand of glucose-free fructose is expected to grow continually. As its commercial production expands and it requires more cost-effective to manufacture. To reduce the cost of manufacture, changing the source of the raw material to fructose-containing polysaccharides, such as inulin is a good choice. This would reduce the number of steps in the production of fructose and is more cost effective^7^.

Jerusalem artichoke can grow in marginal lands and is tolerant to drought and salt stresses^14^. The main carbohydrate in the Jerusalem artichoke tuber is inulin, a fructose-based polymer consisting of linear chains of β-2, 1-linked d-fructofuranose molecules terminated by a glucose residue^15^ that can be easily hydrolyzed into glucose and fructose by inulinase without expensive pretreatment. High fructose syrup (HFS) could be obtained through enzymatic hydrolysis of inulin^16^,^17^. However, it still contains glucose.

*K. marxianus* is a thermo-tolerant non-conventional yeast. It can assimilate some carbon source such as xylose and mannitol which *S. cerevisiae* cannot. Also, *K. marxianus* can directly utilize inulin from Jerusalem artichoke tubers (JAT) at elevated temperature without addition of inulinase and JAT pretreatment^18^,^19^,^20^. This ability makes *K. marxianus* a good candidate to use Jerusalem artichoke tubers at elevated temperatures. As mentioned above, the main mono-sugars in Jerusalem artichoke tubers hydrolysate were glucose and fructose. Glucose-free fructose syrup may be obtained through specific consumption of glucose.

In this study, we identified the genes of hexokinase (*KmHXK1*) and glucokinase (*KmGLK1*) through gene disruption and function analysis. Furthermore, with Jerusalem artichoke tubers, an efficient, thermo-tolerant and one step fructose producing strain was constructed through overexpression of the glucokinase gene in the *KmHXK1* disrupted strain.

## Results

### *KmHXK1* or *KmGLK1* disruption

**Figure 1:**
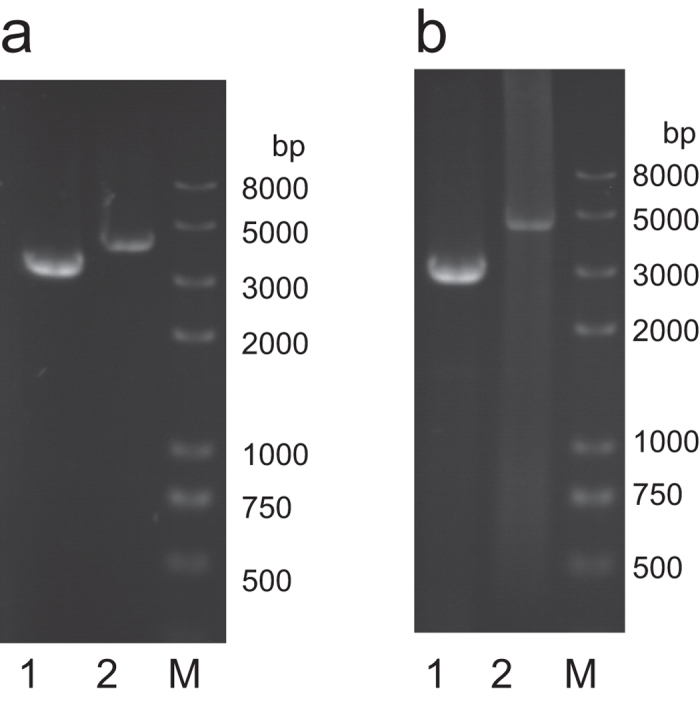
Confirmation of gene disruption by PCR. (**a**) *KmHXK1* and (**b**) *KmGLK1*. 1: YHJ010, 2: YLM001 or YLM002 in a or b respectively.

A hypothetical hexokinase and a hypothetical glucokinase gene were found in the genomic DNA sequence of *K. marxianus*. Through the alignment of amino acid sequences of KmHXK1 and KmGLK1 with other hexokinase (Fig. S1), KmHXK1 (GenBank KX270227) shares 89.4% identity with KlHXK1 (GenBank CAA43855) and 72.4% identity with ScHXK2 (GenBank AAA34699). KmGLK1 (GenBank KX270228) shares 59.6% identity with ScGLK1 (GenBank NP_009890). To identify the function of hypothetical *KmHXK1* or *KmGLK1*, correspondent gene disrupted strains were constructed. After the disruption cassettes containing a *ScURA3* gene were transformed into strain YHJ010 (Table 1) and screened on synthetic dropout (SD) medium without Uracil, the genomic DNA of obtained clones were extracted and used as PCR template. With primer pair KmHXK-F and KmHXK-R or KmGLK-F and KmGLK-R (Supplementary Table S1), a 3.2 Kb (*KmHXK1*) or 2.9 Kb (*KmGLK1*) fragment was amplified from genomic DNA of YHJ010, and a 4.2 kb or a 4.3 Kb fragment was amplified from genomic DNA of YLM001 and YLM002, respectively (Fig. 1). These results indicated a *ScURA3* gene was inserted into *KmHXK1* or *KmGLK1* and proved that they were disrupted (Fig. 1).

### Genes functional evaluation through *KmHXK1* or *KmGLK1* disruption and *KmGLK1* over-expression

**Figure 2:**
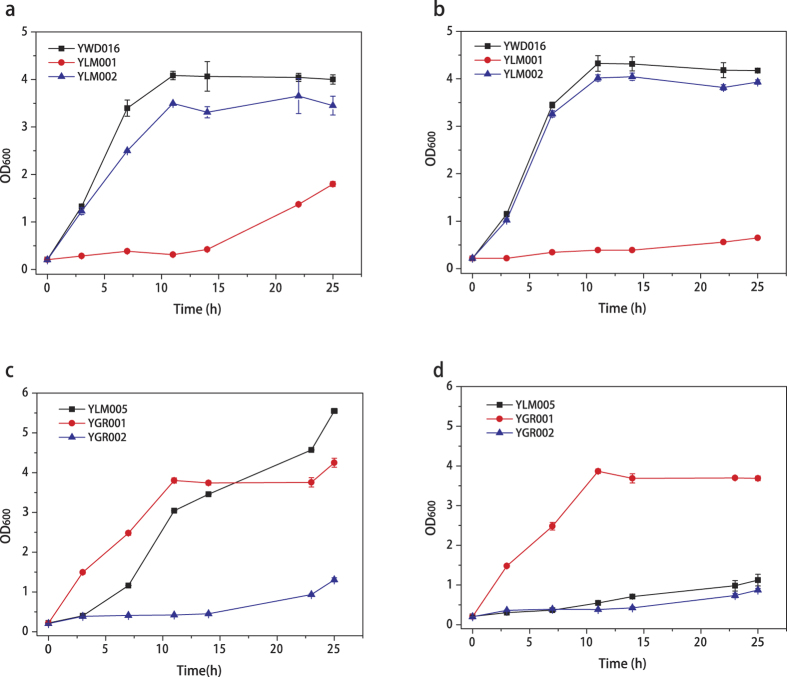
Evaluation of the growth of *KmHXK1* or *KmGLK1* gene disrupted strains with glucose (**a**,**c**) or fructose (**b**,**d**) as carbon source. The growth of YLM001 (*ΔKmHXK1*), YLM002(*ΔKmGLK1*), YLM005 (YLM001 with *KmGLK1* over-expression), YGR002 (YLM001 with *TRP1* complemented) were compared. YWD016 and YGR001 (YHJ010 with *URA3* and *TRP1* complemented) in which auxtrophic markers were complemented were used as empty control (Table 1). The values are the means of three biological replicates ± standard deviation (n = 3).

The strains YLM001, YLM002 and control strain YWD016 which was URA3 complemented strain of YHJ010 (Table 1) were cultivated in SD medium with glucose or fructose as carbon source. The growth of YLM001 decreased significantly with both glucose and fructose (Fig. 2a and b), however, the growth of YLM002 showed little difference with YWD016 (Fig. 2a and b). Therefore, the KmHXK1 was the main enzyme involved in sugar metabolism and it may phosphorylates the hexose during sugar metabolism. However, the function of KmGLK1 was not clear. Then YLM005 which was constructed through over-expressing *KmGLK1* gene in YLM001 was also cultivated with glucose or fructose as carbon source. As shown in Fig. 2c and d, the growth with glucose was recovered, but the growth with fructose was still very weak. These results indicated that KmGLK1 was an enzyme that preferred glucose as substrate.

### Characterization of recombinant KmHXK1 and KmGLK1

To confirm the KmHXK1 and KmGLK1 were glucose or fructose kinase in the hexose metabolism, the recombinant enzymes were characterized. The *KmHXK1* and *KmGLK1* were over-expressed in *Escherichia coli* BL21 (DE3), the recombinant enzymes were purified through His-tag (Supplementary Fig. S2). The kinetic constant of recombinant KmHXK1 and KmGLK1 to glucose and fructose were determined. The *V*max and *K*m of KmHXK1 to fructose are 609.82 U/mg and 0.52 mM, and to glucose are 553.01 U/mg and 0.83 mM respectively. However, the *V*max and *K*m to glucose are 0.73 U/mg and 4.09 mM, and no obvious activity was detected with fructose as substrate.

### Evaluate the sugar consumption of YLM005 strain with glucose-fructose mixture

**Figure 3:**
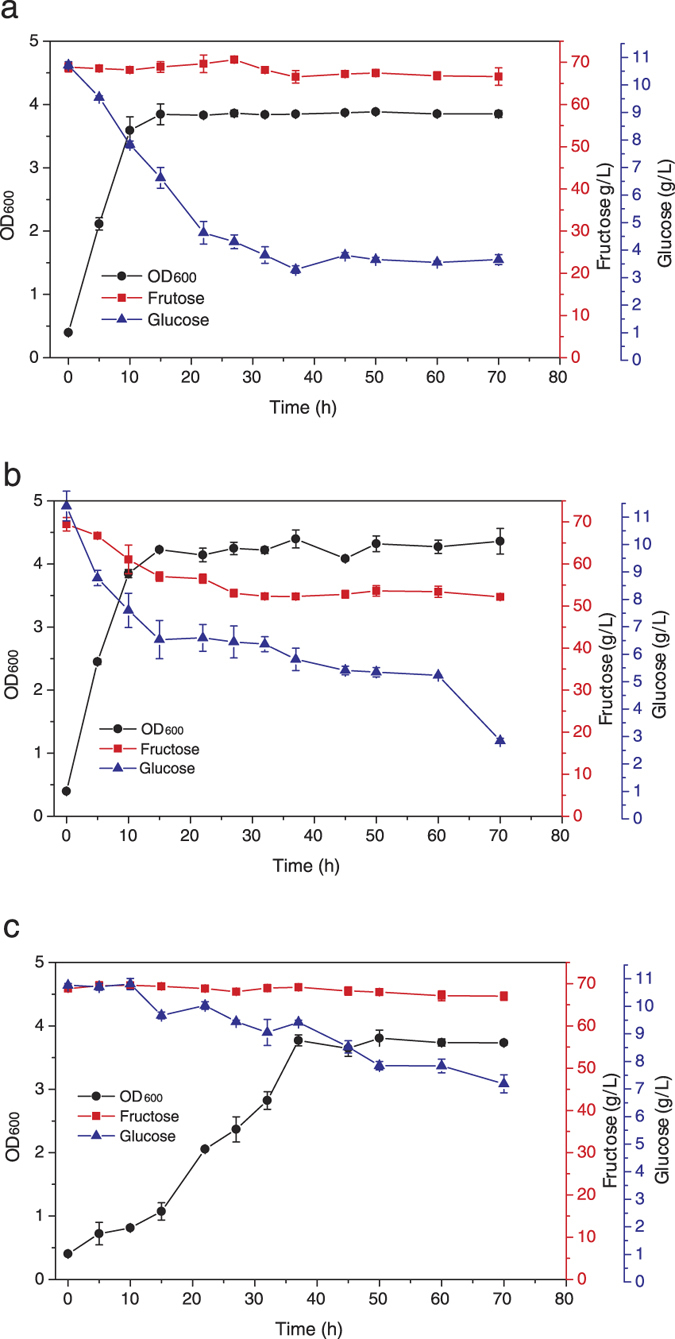
The growth and sugar consumption of constructed strains. YLM005 (**a**), the wild type control YGR001 (**b**) and ΔKmHXK1 control YGR002 (**c**). The values are the means of three biological replicates ± standard deviation (n = 3).

YLM005, control strain YGR001 and YGR002 (Table 1) were cultivated with glucose-fructose mixture (10 g/L and 70 g/L, respectively). YLM005 grew well and reached OD_600_ 3.59 in 10 h. During the growth, the glucose was consumed and decreased to 4.63 g/L in 22 h, however, the fructose was almost not consumed (Fig. 3a). The wild type control strain (YGR001) in which *KmHXK1* and *KmGLK1* was not modified also grew quickly and reached OD_600_ 3.85 in 10 h. However, the glucose and fructose were consumed simultaneously (Fig. 3b). The glucose and fructose concentrations were decreased to 6.45 and 53.07 g/L, respectively. The growth of another strain YGR002, which was derived from YLM001 and *TRP1* auxotrophy was complemented, was poor. It reached OD_600_ 3.77 after 37 h cultivation and only consumed glucose (Fig. 3c). Therefore, in YLM005, the glucose assimilation ability was recovered, while fructose assimilation ability was still defective.

### Hydrolysis inulin and selective consumption of produced glucose by strains YLM005 and YGR003

**Figure 4:**
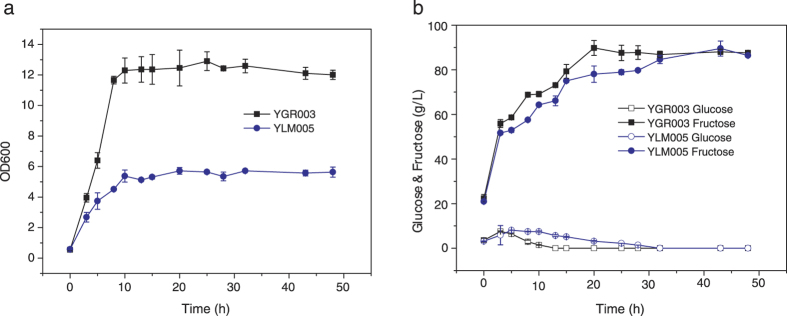
The growth (**a**) and sugar consumption (**b**) of strains YLM005 and YGR003 with 100 g/L pure inulin as carbon source. The values are the means of three biological replicates ± standard deviation (n = 3).

Because *K. marxianus* is a famous inulinase producing yeast, it is possible to obtain a strain which can hydrolyze inulin and produce glucose-free fructose by one step. To improve the inulin hydrolysis ability further, the *LEU2* auxtrophy of YLM005 was complemented by transformation of plasmid pKmLEU^21^ to obtain a non-auxtrophic stain and named YGR003. Then the inulin hydrolysis and glucose consumption ability were evaluated through cultivating the strains with 100 g/L inulin. As shown in Fig. 4a, the growth of YGR003 was faster than YLM005 and could also reach higher cell density. Though the difference was not very large, the glucose consumption and fructose accumulation of YGR003 were stronger than YLM005 (Fig. 4b). Therefore, YGR003 was used in one step glucose-free fructose producing from Jerusalem artichoke tuber (JAT) powder.

### One step producing glucose-free fructose from Jerusalem artichoke tuber powder

Because the pure inulin is relatively expensive material, JAT powder was used as substrate instead. To reduce the cost further, no nutrition except JAT powder and 0.2 M sodium phosphate buffer (pH 5.0) were used in reaction solution. The JAT was bought from the market of Yantai, China and contains 11.27% glucose and 71.10% fructose which were determined through mild acid hydrolysis.

**Figure 5:**
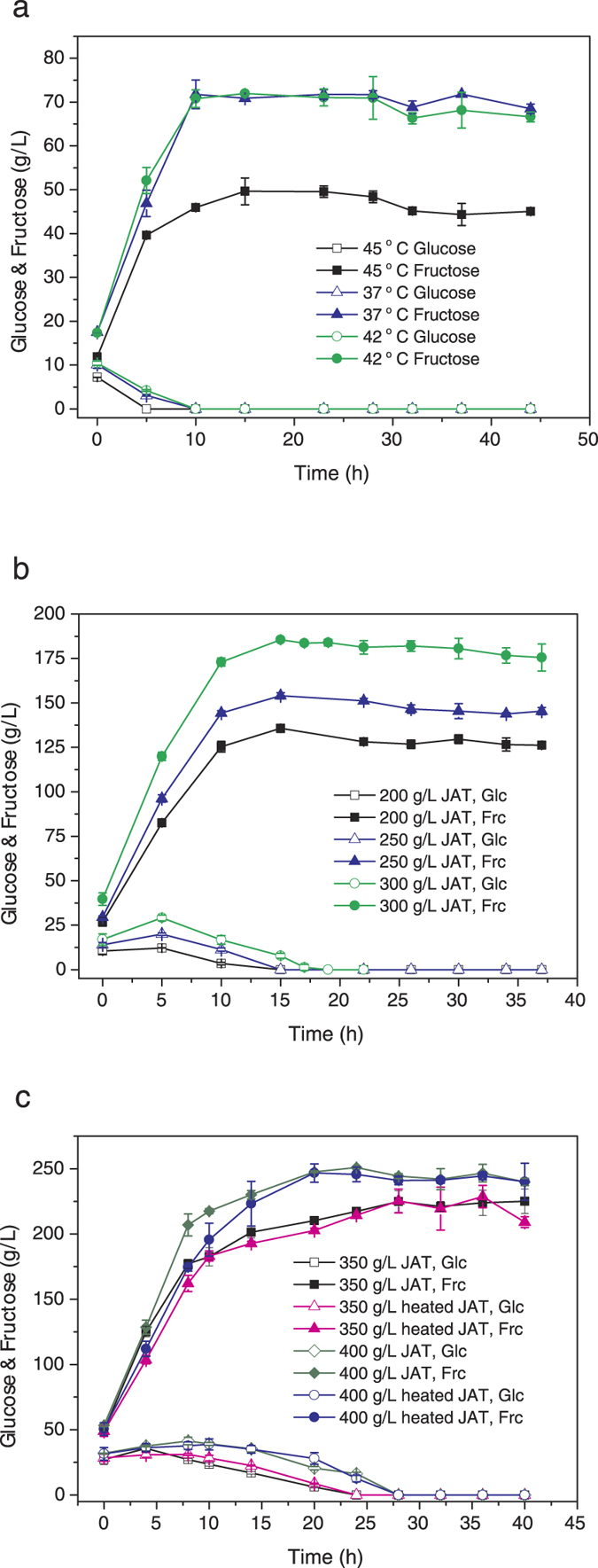
Evaluation of one step producing glucose -free fructose from JAT with strain YGR003. Glucose -free fructose from 100 g/L JAT under various temperature (**a**), with various concentration of JAT (**b**,**c**) at 42 °C, and the effect of heating (**c**) were determined. The values are the means of three biological replicates ± standard deviation (n = 3).

Firstly, the production of glucose-free fructose from 100 g/L JAT powder under various temperatures with YGR003 was evaluated. As shown in Fig. 5a, about 70 g/L fructose was produced in 10 h with a productivity of about 7 g/L/h at 37 and 42 °C. However, at 45 °C, only 45.93 g/L fructose was produced in 10 h (Fig. 5a). Therefore, 42 °C was selected as the temperature for further evaluation with various concentration of JAT powder.

Secondly, the production of glucose-free fructose from JAT powder with various concentrations at 42 °C was evaluated. As shown in Fig. 5 and Table 2, 100, 200, 250, 300, 350 and 400 g/L JAT powder were used. With the JAT concentration increased, the production of fructose was also increased. However, longer time was required to reach glucose depletion. Therefore, the productivity first reached the peak (10.26 g/L/h), then decreased. Though in this study, the concentration of 350 g/L and 400 g/L JAT powder were also evaluated, the mixture of the yeast cells and JAT powder were like thick mud and the mixing efficiency was decreased which led to both the yield and the productivity decrease (Table 2).

Thirdly, producing glucose-free fructose with pre-heated JAT powder was evaluated. In previous reports, pre-heating improved the inulin extraction from JAT^22^. In this study, 350 g/L and 400 g/L pre-heated JAT (80 °C, 90 min) was used to evaluate if the fructose production could be improved further. As shown in Fig. 5c, the fructose produced with pre-heated JAT was not significantly improved. It is reasonable that the hydrolysis procedure took several days, and the release of inulin was not the limitation step.

## Discussion

In this study, the *KmHXK1* and *KmGLK1* genes in *K. marxianus* were experimentally identified. HXK and GLK are the first enzymes in the hexose assimilation pathway after the sugar uptake into cell. After the genomic DNA of *K. marxianus* was sequenced, different to *S. cerevisiae*, only two genes (a *HXK* gene and a *GLK* gene) were found based on the sequence homology. In this study, we confirmed the function of these genes through gene disruption, hexose assimilation analysis and recombinant enzyme characterization. The deficient growth of *KmHXK1* disrupted strain YLM001 with both glucose and fructose as carbon source and the enzymatic activity of recombinant KmHXK1 indicated that KmHXK1 is the main enzyme that catalyzes the hexose phosphorylation. On the other hand, no obvious growth reduction in *KmGLK1* disruption strain YLM002, indicated that *KmGLK1* is not essential in the step of hexose phosphorylation in *K. marxianus*. In our RNA-seq results, the *KmHXK1* expression level was several hundred folds higher than *KmGLK1* (data not shown) in YHJ010 when glucose was the substrate. This result is consistent with above conclusion that KmGLK1 is not the key enzyme in glucose metabolism. The recovered growth on glucose and deficient growth on fructose of *KmGLK1* overexpression strain YLM005 indicated that KmGLK1 is really a glucose kinase. The hexokinase activities in crude lysate of YLM002 to glucose and fructose were 0.82 U/mg and 0.95 U/mg, respectively. The hexokinase activities in crude lysate of YLM005 to glucose and fructose were 0.093 U/mg and 0.067 U/mg. Though the hexokinase activities of YLM005 was very weak, it is strange that the fructose kinase activity in strain YLM005 almost cannot use fructose. Therefore, *KmHXK1* and *KmGLK1* genes were recombinant-expressed and the activities of pure enzymes were determined to avoid background interference. As shown in the results, recombinant KmGLK1 had no obvious fructose kinase activity. On the contrary, KmHXK1 phosphorylates glucose and fructose with relative high activity. The recombinant KmHXK1 has stronger activity to fructose than to glucose which is consistent with the activity in crude supernatant of YLM002. The fructose kinase of YLM005 is possible due to the NADH oxidation by other enzymes in the crude supernatant which was not completely inhibited and led a background. Hexokinase activity was also measured by another method which is through spectrophotometrically detecting optical density at 340 nm (OD_340_) by coupling the reaction to glucose-6-P dehydrogenase (data not shown)^23^. However, no fructose kinase activity could be detected with both KmHXK1 and KmGLK1. It is possible that the glucose-6-P dehydrogenase cannot use fructose-6-p as substrate.

A more convenient glucose-free fructose producing procedure is in great demand. HFCS 42 (42% fructose) is produced from corn starch hydrolysate with glucose isomerase, however HFCS with higher fructose i.e. HFCS 90 was obtained through large scale chromatographic processing of HFCS 42 to remove the glucose^4^,^24^. The preparation of glucose isomerase and large scale chromatography are relative high cost procedure. Inulin contains mainly fructose, it is excellent substrate for high fructose syrup production^17^,^25^,^26^,^27^,^28^,^29^,^30^,^31^. However, inulinase is required in inulin hydrolysis. Though inulinase is widely available, the cost need to be reduced further and preparing and stocking inulinase are also time- and labor-cost procedure in industrial application. There are a lot of microorganism expressing inulinase and hydrolyzing inulin, but the microorganism will utilize the glucose and fructose, leading a decreased yield of high fructose syrup. On other hand, glucose free fructose has a lot of excellent properties. A low cost and simple method for glucose free fructose is required in industrial application. Because YLM005 and YGR003 has strong glucose utilizing ability and very weak fructose utilizing ability, they can specifically consume the glucose in the inulin hydrolysate and the fructose are almost remained unchanged. YLM005 and YGR003 can be used to remove glucose and produce glucose-free fructose syrup from inulin.

Jerusalem artichoke tuber contain 60–80% inulin. Inulin as a reserve carbohydrate is a good feedstock for fructose production. Jerusalem artichoke is easily cultivated. It has high growth rate, good tolerance to frost, drought and poor soil, strong resistance to pests and plant diseases, with minimal to zero fertilizer requirements and the yield of tubers can reach up to 16–20 t/ha for tubers^32^. Furthermore, no nutrition addition is required by using JAT powder to produce fructose since there are some nutrition in JAT powder, and the cost is reduced further. Therefore, JAT is a good feed stock for glucose-free fructose production.

Using constructed yeast strain makes the fructose producing more convenient and efficient. In contrast to expensive enzyme preparation, yeast cell is easy to be stocked, propagated and prepared. Therefore, directly using yeast cell is more convenient than enzyme. Constructing a fructose assimilation deficient and inulin expressing yeast strain should enhance the fructose production from inulin or JAT. Fructose assimilation deficient strains were constructed in *S. cerevisiae* previously through which glucose-free fructose was produced with JAT^29^,^33^. A 8.5% of fructose syrup was obtained through fermentation by *S. cerevisiae* ATCC 36859 with high fructose corn syrup and hydrolyzed JAT juice with a yield of near 100% and a productivity of 1.33 g/L/h^34^. A *HXK* null and inulinase expression *S. cerevisiae* YBS618/INU was constructed. It consumed up to 10% inulin to produce glucose free-fructose. However, the yield and productivity was not indicated. Based on the Fig. 6 in that report, the productivity should be less than 3 g/L/h^33^. Finally, another *HXK* null and inulinase expression *S. cerevisiae* Y16/pHC-PinuT strain produced 9.2% (w/v) glucose free fructose in 24 h with a productivity of 3.83 g/L/h and the yield was not provided^29^. In this study, *K. marxianus* YGR003 used 400 g/L JAT and produced 234.44 g/L fructose with a productivity of 8.37 g/L/h. When 250 g/L JAT is used, the productivity reached 10.26 g/L/h.

Producing fructose with engineered *K. marxianus* has other advantages over *S. cerevisiae*. Because *K. marxianus* is an inulinase producing yeast, no recombinant inulinase expression is necessary. Furthermore, *K. marxianus* is a thermo-tolerant yeast, the fermentation can be conducted at as high as 42 °C. Other than reduction of cool cost, fermentation at elevated temperature can reduce the contamination. In this study, the non-sterilized JAT powder was directly used as substrate and the fructose was still produced efficiently (Fig. 5).

Pre-treating JAT powder is not necessary in this study. In previous report, heating improved the inulin extraction^22^, so the heated JAT powder was also used as substrate to produce glucose -free fructose in this study. However, there is no significant difference between the heated and no-heated JAT powder (Fig. 5c) as substrate. It is possible even without heating, the speed of inulin release matches the speed of hydrolysis already. Therefore, the heating and sterilization of the JAT powder are not necessary in this study.

## Materials and Methods

### Strains and reagents

*K. marxianus* NBRC 1777 was purchased from NITE Biological Resource Center (NBRC, Japan). *K. marxianus* YHJ010 is a *TRP1, LEU2*, and *URA3* auxotrophic strain of NBRC 1777^21^. Yeast Extract Peptone Dextrose (YPD) medium (20 g/L peptone, 10 g/L yeast extract, and 20 g/L d-glucose) was used to aerobically culture *K. marxianus* strains. Yeast Extract Peptone Inulin (YPI) medium (20 g/L peptone, 10 g/L yeast extract, and 20 g/L inulin) was used to grow the seed culture. Synthetic Dropout (SD) medium (6.7 g/L yeast nitrogen base [YNB] without amino acids and 20 g/L d-glucose) supplemented with the appropriate amino acids and/or Uracil was used for transformants selection. Agar plates of all media were prepared by adding 15 g/L agar. All chemical regents used were of analytical grade or higher. Inulin, YNB, d-fructose, d-glucose, and agar were purchased from Sangon Biotech Co. (Shanghai, China). Yeast extract and peptone were obtained from Oxoid (Hants, UK). All modifying enzymes and restriction enzymes were obtained from Thermo Fisher Scientific Co., Ltd. (Waltham, MA). Jerusalem artichoke tubers were purchased from the local market in Yantai, China.

### Disruption of hexokinase and glucokinase gene

A hexokinase and a glucokinase which were named based on homology found in genome of *K. marxianus.* To disrupt hexokinase (*KmHXK1*, Genbank: KX270227) or glucokinase (*KmGLK1*, GenBank: KX270228) gene, the *KmHXK1* and *KmGLK1* gene were amplified from the genomic DNA of *K. marxianus* NBRC1777 with primer pairs KmHXK-F and KmHXK-R or KmGLK-F and KmGLK-R (Supplementary Table S1) respectively. A 3.2 Kb and a 2.9 Kb DNA fragment were obtained and inserted into pGEM-T easy. The resultant plasmid was named as pKmHXK-T and pKmGLK-T (Table 3). Then pKmHXK-T was digested by *Hind* III to remove 1.2 Kb sequence of *KmHXK1* and blunted, pKmGLK-T was digested by EcoR V to remove a 0.8 Kb sequence of *KmGLK1*. A *ScURA3* gene including promoter, open reading frame and terminator was amplified with primers ScURA3-SMAI-FULL-F and ScURA3-SMAI-FULL-R (Supplementary Table S1) using genomic DNA of *S. cerevisiae* W303 1A as temperate. The ScURA3 fragment was digested by *Sma* I and inserted into *Hind* III digested and blunted pKmHXK-T or EcoR V digested pKmGLK-T to obtain plasmid pKmHXK-ScURA3-T and pKmGLK-ScURA3-T respectively (Table 3). Then the *KmHXK1* or *KmGLK1* disruption cassette was amplified with primers KmHXK-F and KmHXK-R or KmGLK-F and KmGLK-R (Supplementary Table S1) and transformed into *K. marxianus* YHJ010 by using a modified lithium acetate method^35^,^36^. The transformants were screened on SD medium without Uracil. After the transformants were obtained, the *KmHXK1* or *KmGLK1* disruption was confirmed by PCR using primer pair KmHXK–F and KmHXK–R or KmGLK-F and KmGLK-R with genomic DNA as temperate. The clones with only one longer product amplified were positive and named YLM001 and YLM002 for *KmHXK1* or *KmGLK1* disruption respectively. *ScURA3* fragment was also transformed into YHJ010 to complement the *URA3* auxotrophic, and the obtained strain was named YWD016 as a non-disruption control (Table 1).

### Over-expression of glucokinase gene

To overexpress *KmGLK1*, the ORF of *KmGLK1* was amplified with primer pair *KmGLK*-F-EcoRI and *KmGLK*-R-NotI (Supplementary Table S1) with pKmGLK-T as template and inserted into YEGAP vector^21^ at *EcoR*I and *Not*I sites. The resultant plasmid was named as YEGAP-GLK (Table 3) which could express *KmGLK1* under *ScTDH3* promoter. Then YEGAP-GLK was transformed into the strain YLM001 in which *KmHXK1* was disrupted and obtained strain was named YLM005. YEGAP was also transformed into YWD016 and YLM001, the resultant strain YGR001 and YGR002 (Table 1) were used as the empty vector control and non-disruption control, respectively. Finally, pKmLeu2 plasmid was transformed into YLM005 to obtain a non-auxotrophic strain YGR003.

### Growth of the constructed *K. marxianus* strains on glucose, fructose and glucose-fructose mixture

To evaluate the sugar assimilation ability of constructed strains, YLM001, YLM002, YLM005 and control strains YWD016, YGR001 and YGR002 (Table 1) were cultivated with glucose or fructose as carbon source. After each strain was precultured in YP-glycerol medium overnight, they were inoculated into SD medium containing 20 g/L glucose or fructose with a starting OD_600_ of 0.2 and aerobically cultivated in a 250-ml flask containing 50 ml of medium at 42 °C with 200 rpm shaking. The growth was assessed by measuring the OD_600_. Furthermore, YLM005 and YGR001 and YGR002 were cultivated in a mixture of 10 g/L glucose and 70 g/L fructose as carbon source. When the strain cultivated on glucose-fructose mixture, other than growth, the sugar consumption was also determined.

### Hydrolysis and utilization of pure inulin with constructed strains

To evaluate the inulin utilization ability of constructed strains, YLM005 and YGR003 were cultivated with SD medium containing 100 g/L inulin after each strain was precultured in YPI medium. Then they were inoculated into SD medium with a starting OD_600_ of 0.2 and aerobically cultivated in a 250-ml flask containing 50 ml of medium at 42 °C with 200 rpm shaking. Other than the growth was determined with OD_600_, the concentration of the glucose and the fructose were also measured.

### One step producing fructose from JAT powder

After YGR003 was confirmed that it can hydrolyze inulin quickly and only utilize glucose, it was used in one step pure fructose production from JAT. The hydrolysis and utilization inulin in 100 g/L Jerusalem artichoke tuber with YGR003 was evaluated. Fifty milliliters fermentation medium containing 100 g/L Jerusalem artichoke tuber powder, 0.2 M sodium phosphate buffer (pH 5.0) was added into a 250-ml flask. Then 5 mL of pre-cultivated yeast cells which were cultivated in 50 ml of YPI and incubated at 37 °C with shaking at 200 rpm for 12 h were inoculated (10%, v/v) and cultivated with 200 rpm shaking. Cultivation temperature of 37, 42, and 45 °C were used to evaluate the effect of temperature on fructose production.

Other than 100, 200, 250, 300, 350 and 400 g/L JAT powder were used to evaluate the fructose production with various substrate concentrations, 80 °C, 90 min heated 350 and 400 g/L JAT powder were used to evaluate if the pre-heating would promote the fructose production. The inulin content in the powder was estimated as described previously^37^. The concentration of glucose and fructose in the culture supernatant during the cultivation were determined by HPLC. All fermentation samples were assayed in triplicate.

### Recombinant expression *KmHXK1* and *KmGLK1* in *E. coli*

The *Nde*I site in *KmGLK1* was removed by silent mutation. Using pKmGLK-T as template, a C- terminal fragment of *KmGLK1* was first amplified by GLKdNdeI-F and KmGLK-XhoI-R (Supplementary Table S1) to eliminate the *Nde*I site. And the N-terminal fragment of *KmGLK1* was amplified by KmGLK-NdeI-F and GLKdNdeI-R (Supplementary Table S1). Then the whole *KmGLK1* with silent mutation was amplified with KmGLK-NdeI-F and KmGLK-XhoI-R through overlap PCR with above two fragments. The *KmHXK1* gene was amplified from pKmHXK-T with primer pair KmHXK-NdeI-F and KmHXK-XhoI-R. Amplified *KmGLK1* and *KmHXK1* were digested by *Nde*I and *Xho*I and inserted into pET22b vector with a His-tag fused to the protein C-terminal. Resultant plasmids were named as pET22b-KmGLK and pET22b-KmHXK (Table 3), respectively. Then the plasmids were transformed into *E. coli* BL21(DE3) to express recombinant enzymes. The expression was induced with 1.0 mM IPTG in LB medium at 37 °C for 4 h when OD_600_ reach 0.5~0.8. After *E. coli* cells were recovered through centrifugation and lysed by sonication, the recombinant enzymes in supernatant of lysed *E. coli* cell were purified by Ni^2+^ charged Chelating Sepharose Fast Flow (GE Healthcare, Uppsala, Sweden) according the product instruction.

### Enzyme activity assay

Hexokinase activity was measured as described previously^38^,^39^ with some modifications. Hexokinase activity to glucose or fructose was assayed in a 50 mM Tris–HCl (pH 7.5) buffered mixture containing glycine (50 mM), KCl (50 mM), ethylenediamine tetraacetic acid (EDTA) (1 mM), NaF (10 mM), phosphoenolpyruvate, tricyclohexylammonium (PEP-Tri) (1 mM), MgCl2 (5 mM), ATP (0.5 mM), NADH (0.3 mM), lactate dehydrogenase (LDH, E.C. 1.1.1.27, 25 U), pyruvate kinase (E.C. 2.7.1.40, 25 U), prepared hexokinase (100 μl), and glucose or fructose (10 mM), and adjusted to a volume of 500 μl with H_2_O. The reaction was initiated by adding 40 mM glucose or fructose, and the activity was determined by monitoring the decrease of absorbance at 340 nm using a spectrophotometer. One unit of D-hexkinase was defined as the amount of enzyme that phosphorylates 1 μmol D-glucose or fructose per min at 28 °C. Protein concentrations were determined via the Bradford method with bovine serum albumin (BSA) as the protein standard^40^.

### Total fructose and glucose quantification

The total fructose and glucose was determined as described previously^41^. 0.2 g of JAT powder was hydrolyzed with 0.2 M HCl at 85 °C for 2 h, and then cooled on ice bath, neutralized with sodium hydroxide, filtered and adjusted to 100 mL with pure water. After 14000 × g centrifugation for 10 min and filtered through 0.2 μm polyethersulfone syringe filter, the samples were analyzed by HPLC.

### HPLC analysis

Glucose and fructose concentrations during fermentation were analyzed by HPLC with a ROA-Organic Acid H+ (8%) column. 2.5 mM H_2_SO_4_ was used as the mobile phase with a flow rate of 0.3 ml/min. The column temperature was 75 °C.

## Additional Information

**How to cite this article:** Guorong, Z. *et al*. Identification of hexose kinase genes in *Kluyveromyces marxianus* and thermo-tolerant one step producing glucose-free fructose strain construction. *Sci. Rep.* **7**, 45104; doi: 10.1038/srep45104 (2017).

**Publisher's note:** Springer Nature remains neutral with regard to jurisdictional claims in published maps and institutional affiliations.

## Supplementary Material

Supplementary Information

## Figures and Tables

**Table 1:** Yeast strains used in this study.

Strain	Relevant genotype	Reference
*K. marxianus* strains		
NBRC1777	Wild type, from NBRC	21
YHJ010	NBRC1777, *ΔKmURA3::*Kan^r^, *ΔKmLEU2::*hisG, *ΔKmTRP1::*hisG	21
YLM001	YHJ010, *ΔKmHXK1::ScURA*3	This study
YLM005	YLM001, YEGAP-*KmGLK1*	This study
YLM002	YHJ010, *ΔKmGLK1::ScURA*3	This study
YWD016	YHJ010, *URA3*	This study
YGR001	YHJ010, YEGAP, *ScURA3*	This study
YGR002	YLM001, YEGAP	This study
YGR003	YLM005, YKmLEU2	This study

**Table 2:** Producing glucose-free fructose with various concentration of JAT powder at 42 °C.

JAT (g/L)	100	200	250	300	350	400
Product (g/L)	70.77 ± 1.99	135.68 ± 2.19	154.03 ± 1.75	183.92 ± 1.84	207.33 ± 2.31	234.44 ± 1.84
Productivity (g/L/h)	7.08 ± 0.20	9.05 ± 0.15	10.26 ± 0.12	9.68 ± 0.01	8.64 ± 0.10	8.37 ± 0.06
Yield* (g/g)	1.00 ± 0.0028	0.96 ± 0.015	0.87 ± 0.010	0.86 ± 0.008	0.83 ± 0.008	0.83 ± 0.006

^*^The yields were calculated based on 71.10% fructose in JAT and calculated as fructose produced (g/L)/fructose added (g/L).

**Table 3:** Plasmids used in this study.

Plasmids	Selection marker and description	Reference
YEGAP	*Amp*^*R*^, *ScTRP*1, P_*KmGAPDH*_, T_*KmGAPDH*_	42
YEUGAP	*Amp*^*R*^, *ScURA*3, P_*KmGAPDH*_, T_*KmGAPDH*_	42
pKmLEU2	*Amp*^*R*^, *KmLEU2*	21
pKmHXK-T	*Amp*^*R*^, *KmHXK1*	This study
pKmHXK-ScURA3-T	*Amp*^*R*^, *KmHXK1* inserted with *ScURA*3	This study
pKmGLK-T	*Amp*^*R*^, *KmGLK1*	This study
pKmGLK-ScURA3-T	*Amp*^*R*^, *KmGLK* inserted with *ScURA*3	This study
YEGAP-KmGLK	*Amp*^*R*^, *ScTRP*1, P_*KmGAPDH*_-*KmGLK*-T_*KmGAPDH*_	This study
pET22b-KmGLK	AmpR, KmGLK expressed under PT7	This study
pET22b-KmHXK	AmpR, KmHXK1 expressed under PT7	This study
