# Supplementary material for: Identification of hexose kinase genes in Kluyveromyces marxianus and thermo-tolerant one step producing glucose-free fructose strain construction
Source: Sci Rep. 2017 Mar 24;7:45104. doi: 10.1038/srep45104 (PMC5364472; doi:10.1038/srep45104)
Supplement: Supplementary Information [file srep45104-s1.pdf]

Supplementary information

Identification of hexose kinase genes in *Kluyveromyces marxianus* and thermo-tolerant one step producing glucose-free fructose strain construction

Zhang Guorong, Lu Min, Wang Jichao, Wang Dongmei, Gao Xiaolian, Hong Jiong*

School of Life Science, University of Science and Technology of China, Hefei, Anhui, 230027, P. R. China

*Correspondence:

Jiong Hong, hjiong@ustc.edu.cn, Telephone: 86-551-63600705, Fax: 81-551-63601443

Table S1 Primers used in this study

Primer	Sequence
KmHXK -F	CATTCCGCTGTCTATTCTAT
KmHXK -R	CAAAGTGGGACTAAGGTTGA
KmGLK-F	TTCTGGTCTGAGGTGGGTCG
KmGLK -R	TGTGATAGTGGCTTCTTCGT
KmGLK-F-EcoRI	CCGGAATTCTTCTGGTCTGAGGTGGGTCG
KmGLK-R-NotI	AATGCGGCCGCTGTGATAGTGGCTTCTTCGT
ScURA3-SMAI-FULL-F	TCCCCCGGGTATTTAGAAAAATAAACAAATAG
ScURA3-SMAI-FULL-R	TCCCCCGGGAATGCGTACTTATATGCGTC
GLKdNdeI-F	GGCGAAGGGTAAGACTCACATGTGCATTAAACT GAATG
GLKdNdeI-R	TGAGTCTTACCCTTCGCC
KmHXK-NdeI-F	GCTCATATGGTTCATTTAGGTCCAAAG
KmHXK-XhoI-R	CCGCTCGAGAGCACCTTCGATACCAACAG
KmGLK-NdeI-F	GCTCATATGTCAGACCCTAAATTAAC
KmGLK-XhoI-R	CCGCTCGAGCAATAAGGCAGCACCGAC

```
KmHXK1     1 MVHLGPKKPPARKGSMADVPADLMEQIHGLETLFTVKTEKMRAIVKHFVSELDKGLSKKG
KlHXK1     1 MVRLGPKKPPARKGSMADVPANLMEQIHGLETLFTVSSEKMRSIVKHFISELDKGLSKKG
ScHXK2     1 MVHLGPKKPQARKGSMADVPKELMQQIENFEKNFTVPTETLQAVTKHFISELEKGLSKKG
KmGLK1     1 -----------------MSDPKLTKAVDEICNQFVIGKSKIAQLTQYFIECMEKGLEPIE
ScGLK1     1 ---------MSFDDLHKATERAVIQAVDQICDDFEVTPEKLDELTAYFIEQMEKGLAPPK
                           △

KmHXK1    61 G--------NIPMIPGWVVESPTGKETG-DFLALDLGGTNLRVVLVKLGGNHDFDTTQNK
KlHXK1    61 G--------NIPMIPGWVVEYPTGKETG-DFLALDLGGTNLRVVLVKLGGNHDFDTTQNK
ScHXK2    61 G--------NIPMIPGWVMDFPTGKESG-DFLAIDLGGTNLRVVLVKLGGDRTFDTTQSK
KmGLK1    44 D--DISTDKGLPMIPTFVTDKPTGQERGTIMLAADLGGTNFRVCSVELLGDHEFKIEQEK
ScGLK1    52 EGHTLASDKGLPMIPAFVTGSPNGTERG-VLLAADLGGTNFRICSVNLHGDHTFSMEQMK

                                                                   *
KmHXK1   112 YRLPDHLRTG----TSEQLWSFIAKCLKDFIEEWYPEGVSE-----PLPLGFTFSYPASQ
KlHXK1   112 YRLPDHLRTG----TSEQLWSFIAKCLKEFVDEWYPDGVSE-----PLPLGFTFSYPASQ
ScHXK2   112 YRLPDAMRTTQ---NPDELWEFIADSLKAFIDEQFPQGISE-----PIPLGFTFSFPASQ
KmGLK1   102 SKIPSSFLQNDPHITSKDLFQHLALVTHQFLNKYHKDQVQN----YKWKMGFTFSYPVDQ
ScGLK1   111 SKIPDDLLDDE-NVTSDDLFGFLARRTLAFMKKYHPDELAKGKDAKPMKLGFTFSYPVDQ
                                                                   △

                        **                                  *
KmHXK1   163 NKINEGVLQRWTKGFDIEGVEGHDVVPMLQEQIKKLNIP-INVVALINDTTGTLVASLYT
KlHXK1   163 KKINSGVLQRWTKGFDIEGVEGHDVVPMLQEQIEKLNIP-INVVRLINDTTGTLVASLYT
ScHXK2   164 NKINEGILQRWTKGFDIPNIENHDVVPMLQKQITKRNIH-IEVVALINDTTGTLVASYYT
KmGLK1   158 TSLKSGNLIRWTKGFQISDTVGKDVVQLFQQELNDIGLSNVHVVALTNDTTGTLLAHCYT
ScGLK1   170 TSLNSGTLIRWTKGFRIADTVGKDVVQLYQEQLSAQGMPMIKVVALTNDTVGTYLSHCYT

                                      *                                 *
KmHXK1   222 DPET-----------KMGIIIGTGVNGAYYESVKNIEKLEGKLPSDITT--DMLMAINCE
KlHXK1   222 DPQT-----------KMGIIIGTGVNGAYYDVVSGIEKLEGLLPEDIGP--DSPMAINCE
ScHXK2   223 DPET-----------KMGVIFGTGVNGAYYDVCSDIEKLQGKLSDDIPP--SAPMAINCE
KmGLK1   218 SSD----EARAISEPVIGCIFGTGTNGCYMEKLDNIHKLDPKIREELKAKGKTHMCINTE
ScGLK1   230 SDNTDSMTSGEISEPVIGCIFGTGTNGCYMEEINKITKLPQELRDKLIKEGKTHMIINVE

                                              *
KmHXK1   269 YGSFDNEHLVLPRTKYDVMIDEQ-SPRPNQQAFEKMTSGYYLGEIMRLVLLDLHSSGFIF
KlHXK1   269 YGSFDNEHLVLPRTKYDVIIDEE-SPRPGQQAFEKMTSGYYLGEIMRLVLLDLYDSGFIF
ScHXK2   270 YGSFDNEHVVLPRTKYDITIDEE-SPRPGQQTFEKMSSGYYLGEILRLALMDMYKQGFIF
KmGLK1   274 WGSFDNELRHLPTTEYDIEIDKKYSANPGYHLFEKRVSGLYLGELLRNILLDMEKKSLID
ScGLK1   290 WGSFDNELKHLPTTKYDVVIDQKLSTNPGFHLFEKRVSGMFLGEVLRNILVDLHSQGLLL

KmHXK1   328 KDQD-----ISKLEVPYVMDTTYPAKIEEDP-FENLEDTYELFKTTLNIETTVIERKLIR
KlHXK1   328 KDQD-----ISKLKEAYVMDTSYPSKIEDDP-FENLEDTDDLFKTNLNIETTVVERKLIR
ScHXK2   329 KNQD-----LSKFDKPFVMDTSYPARIEEDP-FENLEDTDDLFQNEFGINTTVQERKLIR
KmGLK1   334 LKD------SKLSKEPFSLTTETLSDIEVDTFEDGLPTTKDALSKAINWDTTFEQRVVVQ
ScGLK1   350 QQYRSKEQLPRHLTTPFQLSSEVLSHIEIDD-STGLRETELSLLQSLRLPTTPTERVQIQ

KmHXK1   382 KLAELVGTRAARLTVCGVSAICNK-----RGYT-EAHIAADGSVFNKYPGYKEKAAQALK
KlHXK1   382 KLAELVGTRAARLTVCGVSAICDK-----RGYK-TAHIAADGSVFNRYPGYKEKAAQALK
ScHXK2   383 RLSELIGARAARLSVCGIAAICQK-----RGYK-TGHIAADGSVSTRYPGFKEKAANALK
KmGLK1   388 KIVRAISRRAAYLSAVPIAAILIKTNALNKAYHSQVEVGCDGSVVEYYPGFRSMMRHALA
ScGLK1   409 KLVRAISRRSAYLAAVPLAAILIKTNALNKRYHGEVEIGCDGSVVEYYPGFRSMLRHALA

KmHXK1   436 DIYDWKVEKMEDHPIKLVAAEDGSGVGAAIIAALTQKRLAAGKSVGIEGA
KlHXK1   436 DIYNWDVEKMEDHPIQLVAAEDGSGVGAAIIACLTQKRLAAGKSVGIKGE
ScHXK2   437 DIYGWTQPHLDDYPIKVVPAEDGSGPGAAVIAALAQKRIAEGKSVGIIGA
KmGLK1   448 LS---PIGAEGERDVHIRIAKDGSGVGAALCALTADY-------------
ScGLK1   469 LS---PLGAEGERKVHLKIAKDGSGVGAALCALVA---------------
```

Figure S1 Alignment of amino acid sequences hexokinse from *K. marxianus* (KmHXK1), *K. lactis* (KlHXK1) and *S. cerevisiae* (ScHXK2) and glucokinase from *K. marxianus* (KmGLK1) and *S. cerevisiae* (ScGLK1). KmHXK1 (GenBank KX270227) shares 89.4% identity with KlHXK1 (GenBank CAA43855) and 72.4% identity with ScHXK2 (GenBank AAA34699). KmGLK1 (GenBank KX270228) shares 59.6% identity with ScGLK1 (GenBank NP_009890). The underlined sequence of KKPQ/PARKGSM is important for HXK nuclear localization. The underlined conserved sequence PLGFTFS (F/Y) PA existing in several yeast hexokinases. The conserved phosphorylated amino acid Serine 15 and157 of HXK were indicated by open triangle. Glucose binding sites are indicated by asterisks

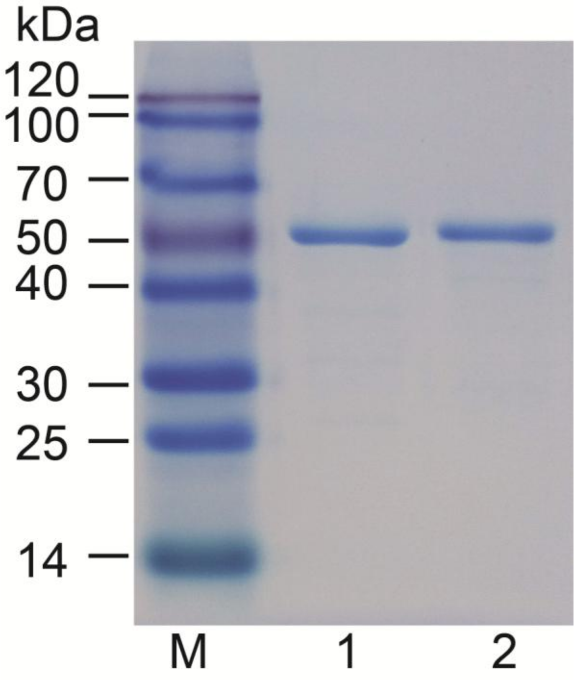

Figure S2 SDS-PAGE analysis of purified recombinant KmHXK1 and KmGLK1. M: molecular weight marker, 1: KmHXK1, 2: KmGLK1.
